# Red vulvar plaque with unilateral edema

**DOI:** 10.1016/j.jdcr.2023.06.017

**Published:** 2023-06-22

**Authors:** Alex Balfour, Jodie Raffi, Bonnie A. Lee, Christina N. Kraus

**Affiliations:** a School of Medicine, University of California, Irvine, California; b Department of Dermatology, University of California, Irvine, Irvine, California

**Keywords:** lymphatic, lymphangioma, ovarian cancer, vulvar

## Case

**Fig 1:**
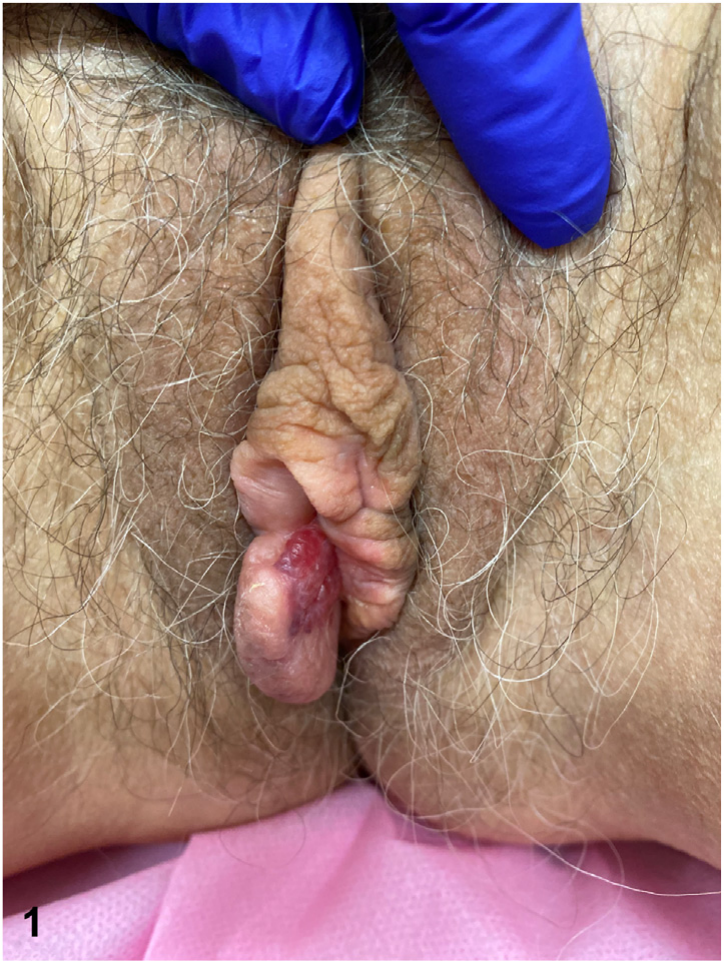

**Fig 2:**
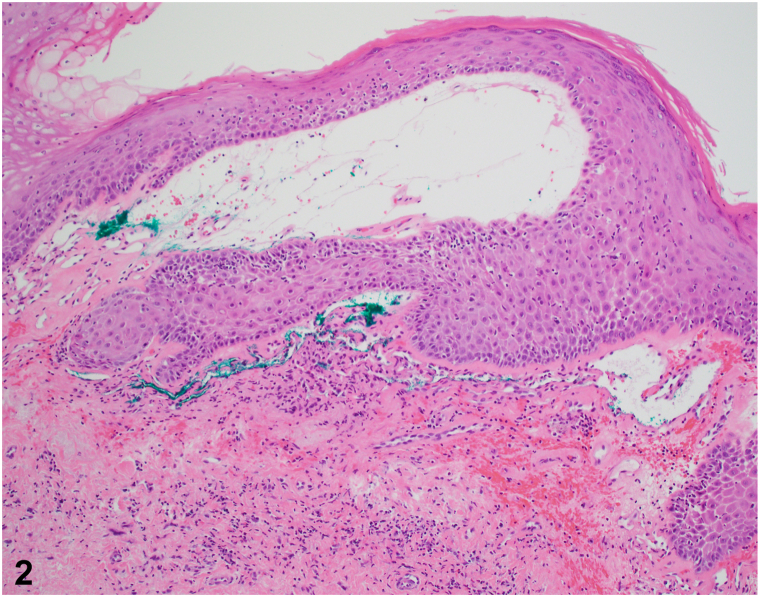

A 75-year-old female presented with right-sided vulvar burning and itch exceeding 1 year. She noticed swelling and tenderness, exacerbated by physical activity, and new onset dyspareunia. She had lichen sclerosus, which was clinically well-controlled with clobetasol. Her medical history was notable for Stage IIIC high-grade serous carcinoma of the bilateral ovaries 9 years prior, treated with cytoreductive surgery, bilateral pelvic lymph node dissection, and chemotherapy. She denied systemic symptoms and had no history of human immunodeficiency virus (HIV) or tuberculosis. On exam, she had a red vesicular plaque with underlying edema (Fig 1) and histological findings are shown (Fig 2).

**Question 1: Which of the following is the most likely diagnosis?**

A. Acquired lymphatic anomaly (ALA)
B. Kaposi sarcoma (KS)
C. Lymphangiosarcoma (LAS)
D. Pyogenic granuloma (PG)
E. Cutaneous endometriosis (CE)

**Answers:**

A. ALA – Correct. Previously known as lymphangioma circumscriptum,^1^ this is a benign dilatation of lymphatic channels, presenting as vesicles or papules, filled with clear or hemorrhagic fluid. ALA is associated with prior pelvic malignancy and related to lymphatic alterations due to surgery or radiation.^2^ Histopathology reveals lymphatic channels without cellular atypia of the luminal cells.
B. KS – Incorrect. KS is a malignant neoplasm of endothelial cells. KS is a red to violaceous plaque, but histopathology would reveal a proliferation of enlarged spindled endothelial cells forming slit-like vessels or irregular vascular spaces surrounding smaller vessels (promontory sign).
C. LAS – Incorrect. LAS is an angiosarcoma associated with chronic lymphedema, with most cases related to prior mastectomy and lymph node dissection (Stewart-Treves).^3^ While this patient has clinical findings that may imitate LAS, biopsy would reveal crack-like dermal spaces with pleomorphic endothelial cells. Some areas may show single cancer cells within the lumen (“school of fish” appearance).
D. PG – Incorrect. PG is a benign vascular proliferation, presenting with rapid growth and bleeding.^4^ A PG would be small and well-circumscribed and not necessarily associated with local edema. Histopathology reveals a proliferation of normal endothelial cells forming tiny vessels in a lobular arrangement.
E. CE – Incorrect. While the vulva is an uncommon location, CE can present as red-brown papules. CE is less common in postmenopausal patients and associated with cyclical pain with menses. Histopathology would reveal extravasated red blood cells and dilated apocrine endometrial glands.

**Question 2: Which of the following is a risk factor for the development of this condition?**

A. Breast cancer
B. HIV
C. Menorrhagia
D. Pregnancy
E. Pelvic malignancy

**Answers:**

A. Breast cancer – Incorrect. A history of breast cancer treated with mastectomy and lymph node dissection, would make Stewart-Treves (lymphangiosarcoma) more likely.^3^ Breast cancer is not a risk factor for ALA.
B. HIV – Incorrect. HIV is a risk factor for AIDS-associated Kaposi Sarcoma. HIV is not a risk factor for ALA.
C. Menorrhagia – Incorrect. Menorrhagia (menstrual bleeding that is heavy and lasts more than 7 days) is associated with endometriosis. Menorrhagia is not a risk factor for ALA.
D. Pregnancy – Incorrect. Pregnancy is a risk factor for PG. Pregnancy is not a risk factor for ALA.
E. Pelvic surgery – Correct. ALA is late-stage complication of anogenital and pelvic malignancies.^2^ The mechanism of ALA is poorly understood, however, it is thought that the disruption of lymphatics through processes like pelvic surgery, lymph node dissection, and radiation can lead to this condition.

**Question 3: Which of the following treatment modalities have been reported with most success for this condition?**

A. Antiretroviral therapy
B. Chemotherapy
C. CO2 laser
D. Hormonal contraceptives
E. Topical timolol gel

**Answers:**

A. Antiretroviral therapy – Incorrect. Antiretroviral therapy would be indicated for AIDS-associated Kaposi Sarcoma. Antiretroviral therapy is not an appropriate therapy for ALA.
B. Chemotherapy – Incorrect. Chemotherapy would be included among treatment options (including surgery and radiation) for lymphangiosarcoma. Chemotherapy is not an appropriate therapy for ALA.
C. CO2 laser – Correct. CO2 devices have been utilized to treat ALA with reported improvement. This device targets more superficial lymphatic vessels, but the deep lymphatic vessels persist, and recurrence is frequent.^5^
D. Hormonal contraceptives – Incorrect. Combined hormonal contraceptives are useful in the treatment of endometriosis. Hormonal contraceptives are not an appropriate therapy for ALA.
E. Topical timolol gel – Incorrect. Topical timolol gel has been utilized to treat some cases of pyogenic granuloma, through antagonism of beta-adrenergic receptors.^4^ Topical timolol is not an appropriate therapy for lymphatic anomalies, such as ALA.

## Conflicts of interest

None disclosed.
